# CCR2^−^ T peripheral helper cells as potential coordinators of local immune architecture in human cancer

**DOI:** 10.1093/discim/kyag007

**Published:** 2026-03-23

**Authors:** Celia del Carmen Crespo Oliva, Zoé Gerber, Dominique Jean, Hugues Allard-Chamard, Marilyne Labrie

**Affiliations:** Centre de Recherche du Centre Hospitalier Universitaire de Sherbrooke (CRCHUS), Sherbrooke, Québec, Canada; Department of Medicine, Division of Rheumatology, Faculty of Medicine and Health Sciences, Cancer Research Institute, Université de Sherbrooke, Sherbrooke, Québec, Canada; Department of Immunology and Cell Biology, Faculty of Medicine and Health Sciences, Cancer Research Institute, Université de Sherbrooke, Sherbrooke, Québec, Canada; Centre de Recherche du Centre Hospitalier Universitaire de Sherbrooke (CRCHUS), Sherbrooke, Québec, Canada; Department of Immunology and Cell Biology, Faculty of Medicine and Health Sciences, Cancer Research Institute, Université de Sherbrooke, Sherbrooke, Québec, Canada; Centre de Recherche du Centre Hospitalier Universitaire de Sherbrooke (CRCHUS), Sherbrooke, Québec, Canada; Department of Immunology and Cell Biology, Faculty of Medicine and Health Sciences, Cancer Research Institute, Université de Sherbrooke, Sherbrooke, Québec, Canada; Centre de Recherche du Centre Hospitalier Universitaire de Sherbrooke (CRCHUS), Sherbrooke, Québec, Canada; Department of Medicine, Division of Rheumatology, Faculty of Medicine and Health Sciences, Cancer Research Institute, Université de Sherbrooke, Sherbrooke, Québec, Canada; Centre de Recherche du Centre Hospitalier Universitaire de Sherbrooke (CRCHUS), Sherbrooke, Québec, Canada; Department of Immunology and Cell Biology, Faculty of Medicine and Health Sciences, Cancer Research Institute, Université de Sherbrooke, Sherbrooke, Québec, Canada

**Keywords:** T peripheral helper cells, tertiary lymphoid structures (TLS), cancer immunotherapy

## Abstract

**Introduction:**

Immune checkpoint inhibitors (ICIs) have revolutionized cancer treatment, yet tumor progression remains a challenge, necessitating improved patient stratification and therapeutic strategies. Tumor growth releases neoantigens that activate adaptive immunity, promoting tertiary lymphoid structure (TLS) formation through B- and T-cell interactions. T follicular helper (Tfh) cells are key in coordinating B-cell responses and are linked to favorable outcomes. A newer subset, T peripheral helper (Tph) cells, shares functional traits with Tfh cells but differs in transcriptional and migratory profiles. Though observed in various cancers, their distribution and role in tumor immunity are not fully understood.

**Methods:**

To investigate this, a multiplex cyclic immunofluorescence assay was developed to detect and spatially analyze Tph cells in tumors.

**Results:**

This revealed three CXCL13-expressing CD4^+^ T-cell subsets: Tfh, Tph, and a ‘C-C motif chemokine receptor 2 (CCR2)^−^ Tph-like’ population which, in contrast to the classically CCR2-enriched Tph phenotype described in inflamed tissues, shows markedly reduced CCR2 expression. Downregulation of CXCR5 and CCR2 near CCR2^−^ Tph-like cells suggested a shift toward local immune residency, forming immune niches. These niches were enriched with pro-inflammatory cells, including Th1, Th17, CD4^+^, CD8^+^ T cells, and B cells. Spatial profiling showed CCR2^−^ Tph-like cells embedded in an immunoregulatory network, marked by CD69 and inhibitory checkpoints B7-H3 and PD-L1 on surrounding cells.

**Conclusions:**

This dual signaling suggests CCR2^−^ Tph-like cells may modulate tumor immunity by balancing activation and suppression, with potential implications for checkpoint blockade therapy.

## Graphical Abstract

**Graphical Abstract:**
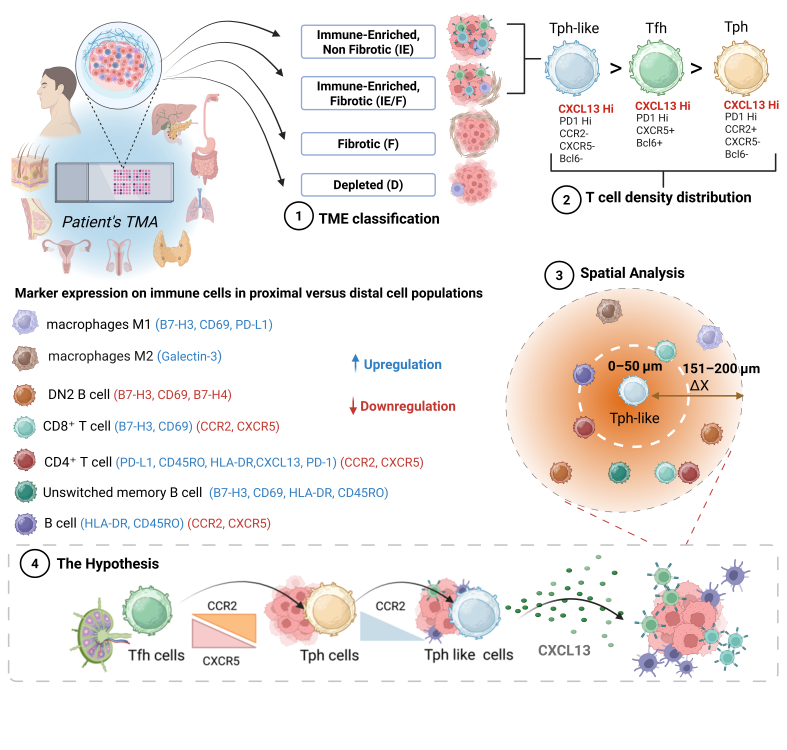
Graphical abstract created in BioRender. Labrie, M. (2026) https://BioRender.com/iymn00y

## Introduction

Immunotherapies like immune checkpoint inhibitors (ICIs) based on anti-programmed cell death protein 1(PD-1)/programmed death-ligand 1 (PD-L1) and anti-cytotoxic T-lymphocyte-associated protein 4 (CTLA4) have revolutionized cancer patient care. Nearly half of all cancer patients have a tumor seemingly compatible with immunotherapy [1]. Yet, the current response rate to ICIs is only 15%, posing a significant challenge in accurately identifying patients most likely to benefit from these treatments [2]. Additionally, up to 40% of patients exposed to ICIs experience adverse immune-related events (irAEs), potentially leading to chronic inflammation that persists even after discontinuing the therapy [3]. To achieve higher remission rates and prevent unnecessary treatments that would lead to severe irAEs, it is crucial to better understand immunotherapy mechanisms and how their interplay with the immune system leads to long-term immune-mediated side effects [4]. Recent studies have shown that various pre-existing or acquired traits can impact the effectiveness of ICIs, including the development of tertiary lymphoid structures (TLSs) in the vicinity of cancer tissue [5]. TLSs are ectopic lymphoid organs that form in non-lymphoid tissues in response to chronic inflammation [6]. Tumor development is associated, in several cases, by the activation of immune pathways leading to sustained levels of inflammation, thus nurturing a milieu supporting the local development of TLSs [7]. These structures, which mature to form germinal centers, are organized with distinct areas enriched in B and T cells, suggesting a role in initiating and sustaining a strong adaptive immune response encompassing humoral and cellular antitumor immune responses [8]. Indeed, mature TLSs contain mature B cells and produce tumor-specific antibodies that are associated with improved prognoses in cancer patients [9].

Before 2019, substantial evidence suggested that CXCL13-secreting T follicular helper (Tfh) cells (BCL6^+^, CXCR5^+^, PD-1^+^) were key contributors to TLS organization and B-cell recruitment, and several studies proposed that these cells can promote TLS formation in tumors and chronic inflammatory settings [10, 11]. Recently, it was demonstrated that a new subset of T peripheral helper (Tph) cells also might play a role in forming these structures [10, 12]. Tph cells, defined as PD-1^+^, CXCR5^−^, Bcl6^low/−^, CD4^+^ T cells exhibit variable expression of chemokine receptors, including C-C motif chemokine receptor 2 (CCR2), which influences their ability to shape the tissue microenvironment and guide B-cell infiltration into peripheral tissues. Previous studies show that CCR2 expression is preferentially enriched in CXCR5^−^ Tph cells, while being largely absent from CXCR5^+^ Tfh cells; however, CCR2 is not uniformly expressed across all Tph populations, and its prevalence appears to vary by disease context [13, 14].

Transcriptomic analyses have revealed significant overlap in gene signatures between Tfh and Tph cells, including macrophage activating factor (MAF), T cell immunoreceptor with Ig and ITIM domains (TIGIT), and signaling lymphocyte activation molecule family member 6 (SLAMF6), indicating potential functional similarities or convergent pathways. Both Tph and Tfh cells support plasma cell differentiation by engaging with interleukin (IL)-21 and SLAMF5 [15]. While B cells are known to play crucial roles in antitumor immunity [16], the contribution of Tph cells to shaping the cancer microenvironment remains incompletely understood. In particular, how the spatial localization and functional specialization of CCR2^+^ versus CCR2^−^ Tph subsets may drive nonredundant roles within tumor niches is still an open question with important implications for understanding T-cell–B-cell cross-talk in cancer. Recently, the potential significance of CXCL13-producing Tph cells in various cancers, including ovarian cancer (OV) [10], nasopharyngeal carcinoma (NPC) [12], and breast cancer (BC) [11], has been highlighted [17]. These Tph cells contribute to the antitumor immune response by facilitating TLS formation and supporting B-cell-mediated antitumor antibody responses or B cells acting as local antigen-presenting cells. Tph cell–induced structures may thus enhance the local antitumor immune response, though further research is needed to fully elucidate their role and impact across different cancer types [17].

Differentiating responders from nonresponders to immunotherapy remains challenging due to the complexity of the immune response and tumor heterogeneity [18]. Proposed biomarkers, including PD-L1 expression [19], CD8^+^ T-cell infiltration [20], tumor mutational burden [18], inflammatory gene signatures (e.g. interferon [IFN]-γ) [21], and immune-infiltrated tumor microenvironments (TMEs) [22]. These predictors, however, are insufficient for precise patient treatment stratification. Critical gaps remain in understanding immune cell spatial organization and immunoregulatory interactions within tumors [23]. Given their role in anticancer immune activation, Tph cells may be valuable biomarkers for predicting immunotherapy responses. However, there is currently no standardized approach for their detection in cancer tissues.

The majority of Tph cell characterization has relied on flow cytometry, a technique that, while powerful, precludes the analysis of cellular interactions within the native tissue architecture [24]. Recent advancements in proteomics, particularly multiplex antibody-based assays, have significantly improved our understanding of the cellular network organization within the TME [25]. Building on these developments, we have developed a cyclic immunofluorescence (Cyc-IF) method to detect Tph cells and spatially characterize the immune landscape of cancer tissues infiltrated by Tph cells [26]. Our study has allowed us to identify a different cell state within the Tph cells spectrum (CCR2^−^ Tph-like cells). Unlike Tfh and conventional Tph cells, CCR2^−^ Tph-like cells are broadly distributed across tumors, particularly within immune-enriched microenvironments. These findings suggest CCR2^−^ Tph-like cells could be involved in the reshaping of the tumor immune microenvironment, representing a promising biomarker for immunotherapy.

## Materials and methods

### Tissue microarray and clinicopathological data

**Figure 1:**
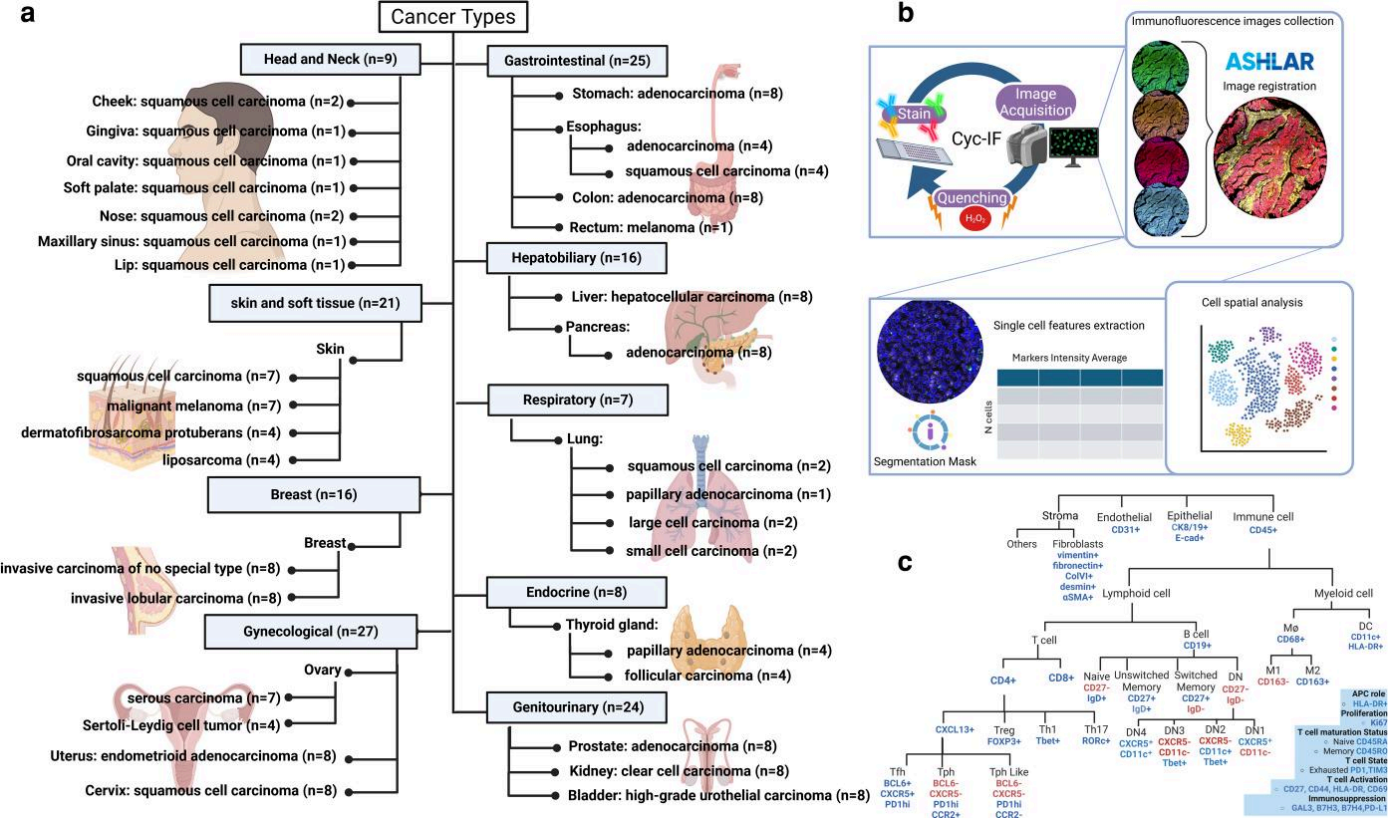
Detection of Tph-related cells by Cyc-IF. (a) Comprehensive cancer subtype classification framework. (b) Schematic of Cyc-IF methodology demonstrating iterative cycles of immunofluorescence staining, high-resolution imaging, and signal quenching on individual tissue slides. Workflow includes precise image alignment, automated cell segmentation, and quantitative extraction of marker intensities for spatially resolved single-cell characterization (c) Custom multicolor antibody panel designed to comprehensively identify diverse immune cells phenotypes across tissue microenvironments. Figure 1 created in BioRender. Labrie, M. (2026) https://BioRender.com/fn3zwdw.

A pan-cancer tissue microarray (TMA) (MC2082D) was obtained from TissueArray.com LLC (formerly US Biomax). It features 5-µm-thick sections with 1-mm core diameters from formalin-fixed, paraffin-embedded tissues. Each case is represented by a single core encompassing samples from various untreated cancer types. Each cancer type was classified into nine subgroups based on organ systems and histological types, following the World Health Organization (WHO) classification of tumor series [27, 28] and the International Classification of Diseases for Oncology (ICD-O) [28] (Fig. 1a). Due to technical limitations, we focused our analysis on solid tumors and excluded the central nervous system (CNS), germ cell tumors, and hematological malignancies. Samples and patients’ information are provided in Supplementary Table S1.

### Antibody selection and validation

A comprehensive list of commercial primary antibodies used in this study is provided in Supplementary Table S2. For unconjugated antibodies, in-house antibody labeling was performed using Alexa Fluor conjugation kits (Invitrogen): Alexa Fluor 488 (Cat. A37570), Alexa Fluor 555 (Cat. A37571), Alexa Fluor 647 (Cat. A37573), and Alexa Fluor 750 (Cat. A37575), following the manufacturer’s protocols. Antibody validation was performed by staining a control TMA comprising lymphoid tissues as well as normal and malignant tissues of various origins. For each antibody, the staining patterns across tissue types, including both positive and negative signals, were systematically compared to reference data from the Human Protein Atlas and the published literature, ensuring specificity and concordance with established expression profiles. Additionally, we have included Supplementary Figure S1, which demonstrates the staining pattern of CCR2 in human tonsil tissue, confirming consistency with previously reported data.

### Cyc-IF

Cyc-IF was performed as previously described [29]. Briefly, the TMA was deparaffinized, and antigen retrieval was conducted using a Cuisinart pressure cooker (model CPC-600) with pH 6 citrate buffer for 20 minutes. The slides were then rinsed in distilled water and incubated in warm pH 9 Tris/ethylenediaminetetraacetic acid (EDTA) buffer for 15 minutes. Next, the slides were blocked in phosphate-buffered saline (PBS), containing 10% normal goat serum and 1% bovine serum albumin (BSA). Tissue autofluorescence was captured using an Axioscan 7 fluorescence slide scanner (Zeiss). Sequential staining, imaging, and quenching processes were then performed for a total of 17 cycles. Each cycle included incubation of the slides with four primary antibodies conjugated to Alexa-Fluor 488, 555, 647, or 750, or their equivalent, followed by imaging using the Axioscan 7 fluorescence slide scanner. The fluorescence signals were then quenched in a solution of 3% hydrogen peroxide and 20 mM NaOH in PBS for 30 minutes. The quenching of immunofluorescence signals was verified before proceeding to the next set of antibodies, until all antibodies were probed.

### Image and single-cell data processing

The image analysis pipeline integrated established tools and custom Python scripts to process the acquired raw images (Fig. 1b). We used ASHLAR (Alignment by Simultaneous Harmonization of Layer/Adjacency Registration) for image registration, a methodology available on GitHub (https://github.com/labsyspharm/ashlar). For multiplex image visualization, cell segmentation, and feature extraction, we employed QI Tissue Image analysis software version 1.4.0 (Quantitative Imaging Systems). The analysis workflow included extracting the mean intensities of each marker from appropriate cell compartments, filtering cells with abnormal features based on nucleus size and autofluorescence levels, and subtracting autofluorescence levels for each marker on a single-cell basis. Finally, protein expression values were normalized using *z*-score calculations to enable comparative analysis across samples and markers, as previously described by our group [29] (github: https://github.com/biodev/cycIF-workflow/tree/v1.0). After data normalization, each cell was attributed to a cell type based on its marker expression (Fig. 1c).

### Data analyses and visualization

Computational and statistical analyses were implemented in Jupyter Notebook (version 7.0.8) using Python (version 3.10.14) via Anaconda distribution. The analytical pipeline utilized specialized Python libraries: data manipulation and analysis were performed with Pandas (version 1.5.3) and NumPy (version 1.24.3), while statistical computations were executed using SciPy (version 1.14.0) and StatsModels (version 0.14.2). Data visualization was performed by Matplotlib (version 3.7.5) for figures and Seaborn (version 0.11.2) for statistical plots, enabling the creation of publication-quality graphics, including boxplots, dot plots, and statistical annotations. For spatial analyses, the Euclidean distance between target immune cell subtypes and their nearest reference cell populations was measured using the spatial coordinates of the cells extracted during segmentation.

### Statistical analysis and figures

All statistical analyses were conducted using Python (version 3.10.14) within Jupyter Notebook (version 7.0.8) via the Anaconda distribution. Statistical computations were performed using SciPy (version 1.14.0) and StatsModels (version 0.14.2). Categorical variables with multiple groups were analyzed using Chi-square tests, while continuous variables between groups were evaluated through nonparametric Mann–Whitney *U* tests and Wilcoxon signed-rank tests for paired comparisons. Statistical significance was established using standardized threshold conventions: not significant (ns) for *P* ≥ 0.05, * for *P* < 0.05, ** for *P* < 0.01, *** for *P* < 0.001, and **** for *P* < 0.0001. Box plot visualizations were standardized with whiskers extending to either the maximum/minimum values or 1.5 times the interquartile range (IQR) above the 75th and below the 25th percentiles, ensuring consistent and precise data representation. Schematic diagrams were created using BioRender.

## Results

### CXCL13-positive CD4 T cell populations are found across cancer types

**Figure 2:**
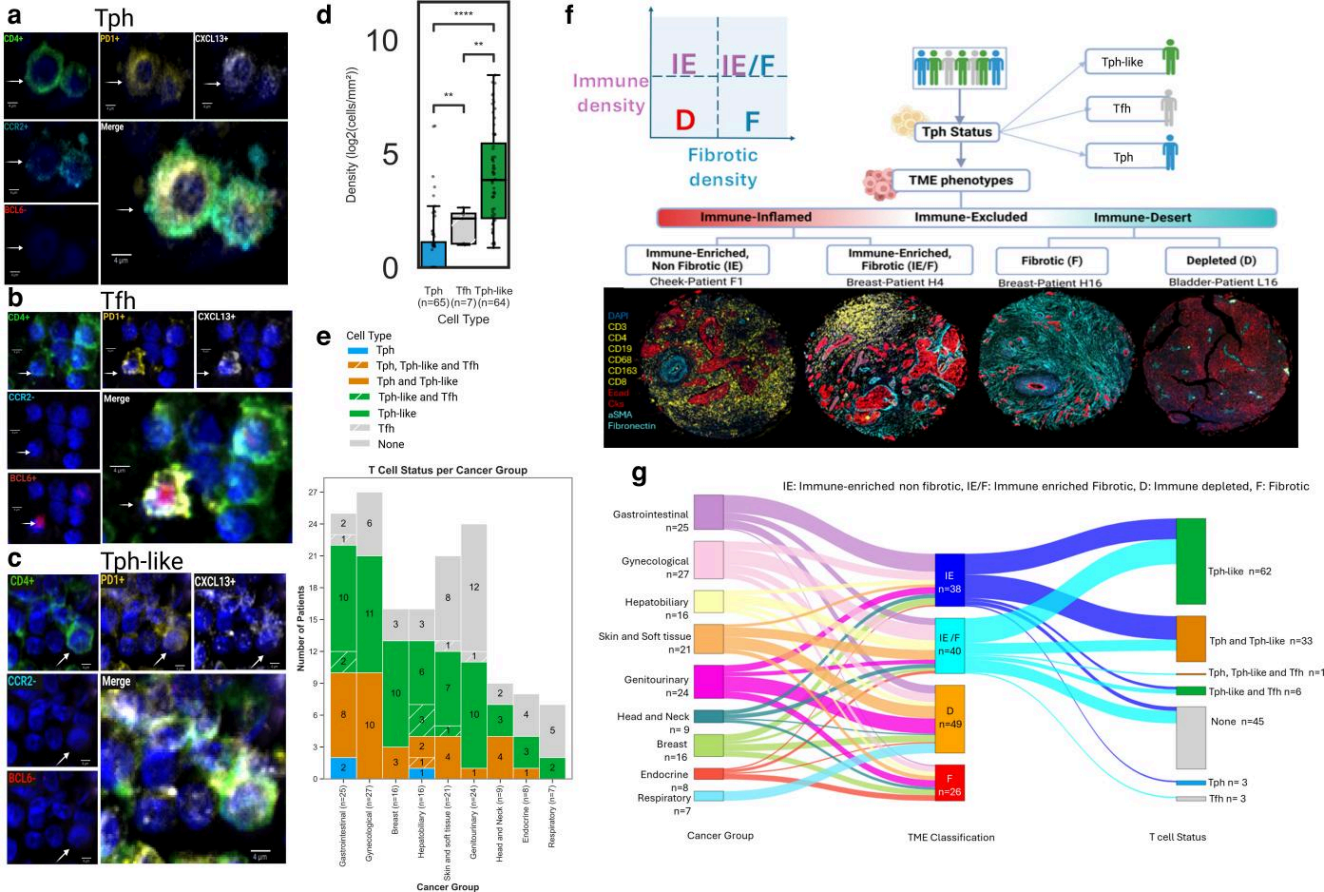
Spatial characterization of Tph, Tfh, and Tph-like cells across tumor microenvironments. Representative cellular phenotype identification across different tissue contexts: (a) T peripheral helper (Tph) cells in ovarian tissue (Patient I4) are CD4+, PD1+, CCR2+, CXCL13+, CXCR5-, Bcl6-), (b) T follicular helper (Tfh) cells in oral cavity (Patient F4) are CD4+, PD1+, CCR2-, CXCL13+, CXCR5+, Bcl6+, and (c) CCR2^−^ T peripheral helper-like (hereafter referred to as Tph-like) cells in breast tissue (Patient H4) are CD4+, PD1+, CCR2-, CXCL13+, CXCR5-, Bcl6-. Marker colors: CD4 (green), PD1 (yellow), CXCL13 (white), CCR2 (cyan), BCL6 (red). (d) Density distribution of T cell subtypes across cancer subsets. Statistical analysis performed using Mann-Whitney U test with Benjamini-Hochberg false discovery rate correction. Significance levels: ns = not significant, * = *P* < 0.05, ** = *P* < 0.01, *** = *P* < 0.001, **** = *P* < 0.000. (e) Patient-level identification of infiltrating Tph, CCR2^−^ Tph-like, and Tfh cell populations. (f) Immunogram schematic illustrating patient stratification based on T cell status and TME phenotypes. Lower panel: Representative multiplex immunofluorescence images demonstrating four distinct TME classifications: Immune-Enriched Non-Fibrotic (IE), Immune-Enriched Fibrotic (IE/F), Fibrotic (F), and Depleted (D). (g) Sankey diagram depicting the relationship between cancer types (left), TME classifications (middle), and T cell status (right). Flow widths represent proportional distributions across multiple cancer types, including gastrointestinal, gynecological, hepatobiliary, head and neck, genitourinary, skin and soft tissue, breast, endocrine, and respiratory cancers. Figure 2 created in BioRender. Labrie, M. (2026) https://BioRender.com/7xvphy1.

Tph cells have been identified as a distinct subset of CXCL13-expressing CD4^+^ T cells that play a crucial role in immune cell recruitment and local immune organization [12, 30]. We used Cyc-IF to identify and quantify the density of Tph cells across cancer types. Tph cells were identified by the co-expression of CD4, PD1, CCR2, and CXCL13, and the absence of CXCR5 and Bcl6 (Fig. 2a). This specific marker combination differentiated Tph cells from other T-cell subsets, such as Tfh cells (CD4+, PD1+, CXCL13+, Bcl6+, CXCR5+) (Fig. 2b). Interestingly, this nuanced approach allowed us to find a cell population closely related to the profile of Tph cells, except for their lack of CCR2 expression (CD4+, PD1+, CXCL13+, CCR2-, CXCR5-, Bcl6-) (Fig. 2c). To our knowledge, cells with this combination of immune markers have not been previously characterized. Due to their close similarity to Tfh and Tph cells, CCR2^−^ Tph-like cells were included in the downstream analyses. Comprehensive analysis across multiple cancer subtypes revealed that CCR2^−^ Tph-like cells were commonly found across tumors with a median density of 22.84 cells/mm^2^ and were significantly more abundant than Tfh cells (density of 4.90 cells/mm^2^, *P* < 0.01) and Tph cells (density of 1.80 cells/mm^2^, *P* < 0.0001) (Figs 2d and e). While mixed infiltration patterns of Tph and CCR2^−^ Tph-like cells were observed in some tissues, exclusive Tfh or Tph presence was relatively rare, suggesting the co-migration of these cells in the tissues (Fig. 2e).

### CXCL13+ CD4+ T cells are enriched in immune-inflamed tumors

Given the exploratory, pan-cancer design of our TMA approach, which was intended to maximize the detection of CCR2^−^ Tph-like cells across a broad range of malignancies, while acknowledging the limited sample sizes per cancer type, we systematically grouped tumors into higher-order categories using current WHO/ICD-O definitions. CCR2^−^ Tph-like cells were quantified and visualized across these consolidated cancer groups and further stratified by TME phenotype. This strategy allowed us to transcend conventional histological distinctions and analyze the immune context specifically in the vicinity of CCR2^−^ Tph-like cells. To examine the immune landscape of tumors infiltrated by Tph, CCR2^−^ Tph-like and Tfh cells, we classified each patient’s TME using the framework established by Alexander *et al* [22]. In their study, they identified four distinct TME profiles predictive of immunotherapy response. These classifications include immune-enriched (IE), immune-enriched fibrotic (IE/F), fibrotic (F), and immune-desert phenotypes (D), each defined by differences in immune cell infiltration, stromal composition, and spatial immune organization. Their findings emphasize the pivotal role of immune–TME interactions in shaping treatment outcomes, with immune-enriched phenotypes demonstrating the greatest responsiveness to ICIs, whereas immune-excluded and immune-desert TMEs are linked to poor responses due to immune evasion mechanisms [22]. Multiplexed imaging confirmed the presence of four distinct TME phenotypes, each defined by varying levels of immune infiltration, ranging from immune-inflamed to immune-desert states (Fig. 2f). Sankey diagram analysis revealed distinct distribution patterns of Tph, Tfh, and CCR2^−^ Tph-like cells across cancer types, with CCR2^−^ Tph-like cells notably enriched in immune-inflamed TME phenotypes (Fig. 2g). Among these, 89.5% of IE-classified samples and 75% of IE/F-classified patient samples exhibited CCR2^−^ Tph-like cell infiltration (Supplementary Figure S2a and b). Taken together, these results indicated a stronger correlation between immune-enriched TME and CCR2^−^ Tph-like cells compared to Tph and Tfh cells.

Collectively, these results indicate that CCR2^−^ Tph-like cell infiltration is not limited to any single tumor lineage but instead represents a consistent feature of immune-inflamed TMEs across diverse malignancies. This pattern underscores the potential of CCR2^−^ Tph-like cells and their pathways as broad therapeutic targets, irrespective of cancer histotype.

### The spatial organization of CCR2^−^ Tph-like cells defines immune-enriched niches in the tumor microenvironment

**Figure 3:**
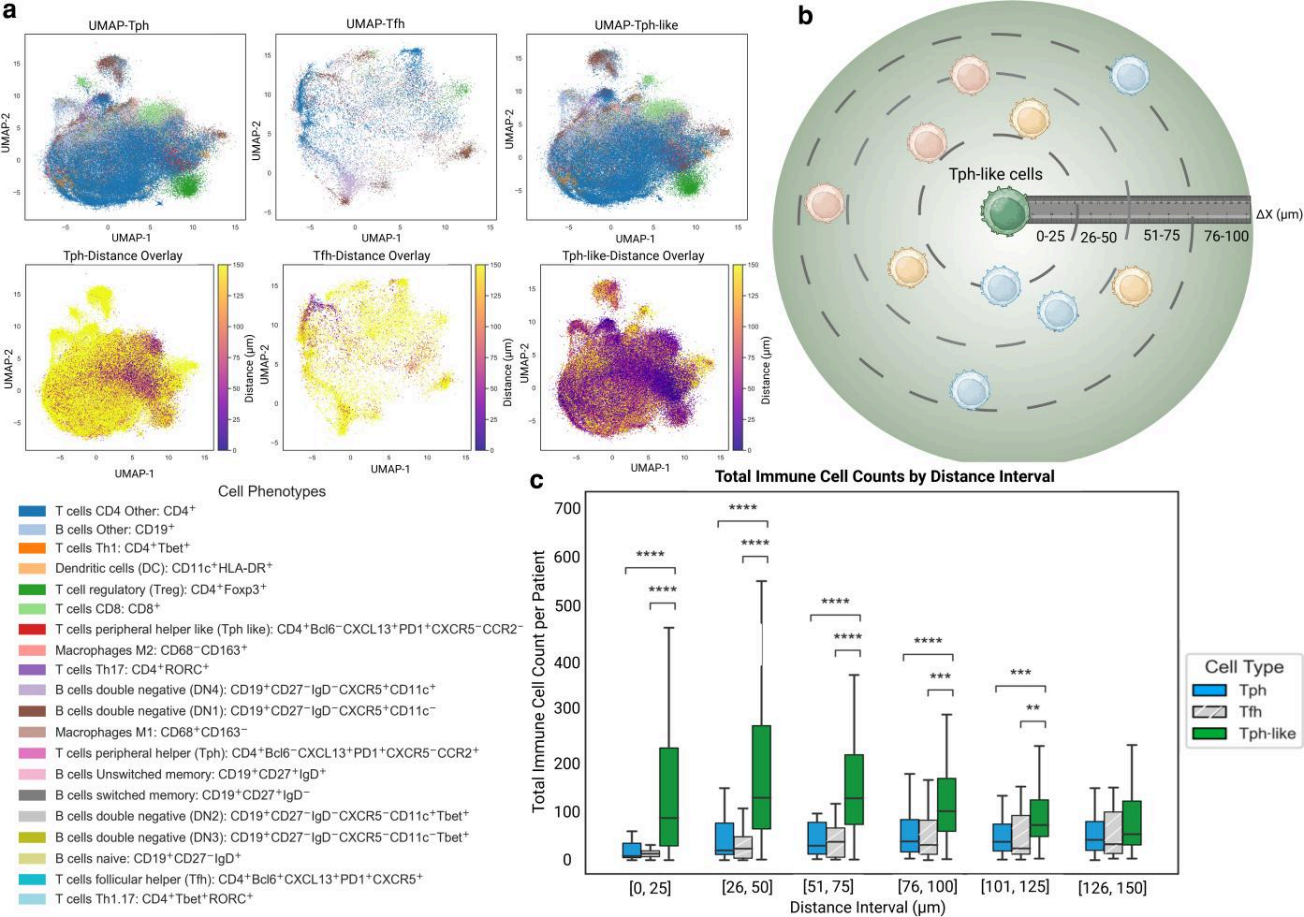
CCR2^−^ Tph-like cells act as immune hubs, structuring spatial organization in the tumor microenvironment (a) UMAP-based clustering and spatial mapping of immune cell subtypes, highlighting distinct clustering of immune cell subpopulations and distance-based spatial overlays for Tph, Tfh, and CCR2^−^ Tph-like cells.(b) Cartoon illustrating the spatial analysis framework, using CCR2^−^ Tph-like cells as reference points to quantify immune cell densities across increasing radial distance zones.(c) Quantitative spatial analysis of immune cell distribution, measured across 25 μm intervals (0–25, 25–50, 50–75, 75–100 μm) relative to reference populations (Tfh, Tph, and CCR2^−^ Tph-like). Statistical comparisons were performed using the Mann-Whitney U test with Benjamini-Hochberg false discovery rate correction. Significance levels: ns = not significant, * *P* < 0.05, ** *P* < 0.01, *** *P* < 0.001, **** *P* < 0.0001. Figure 3 created in BioRender. Labrie, M. (2026) https://BioRender.com/bqxm1pj.

**Figure 4:**
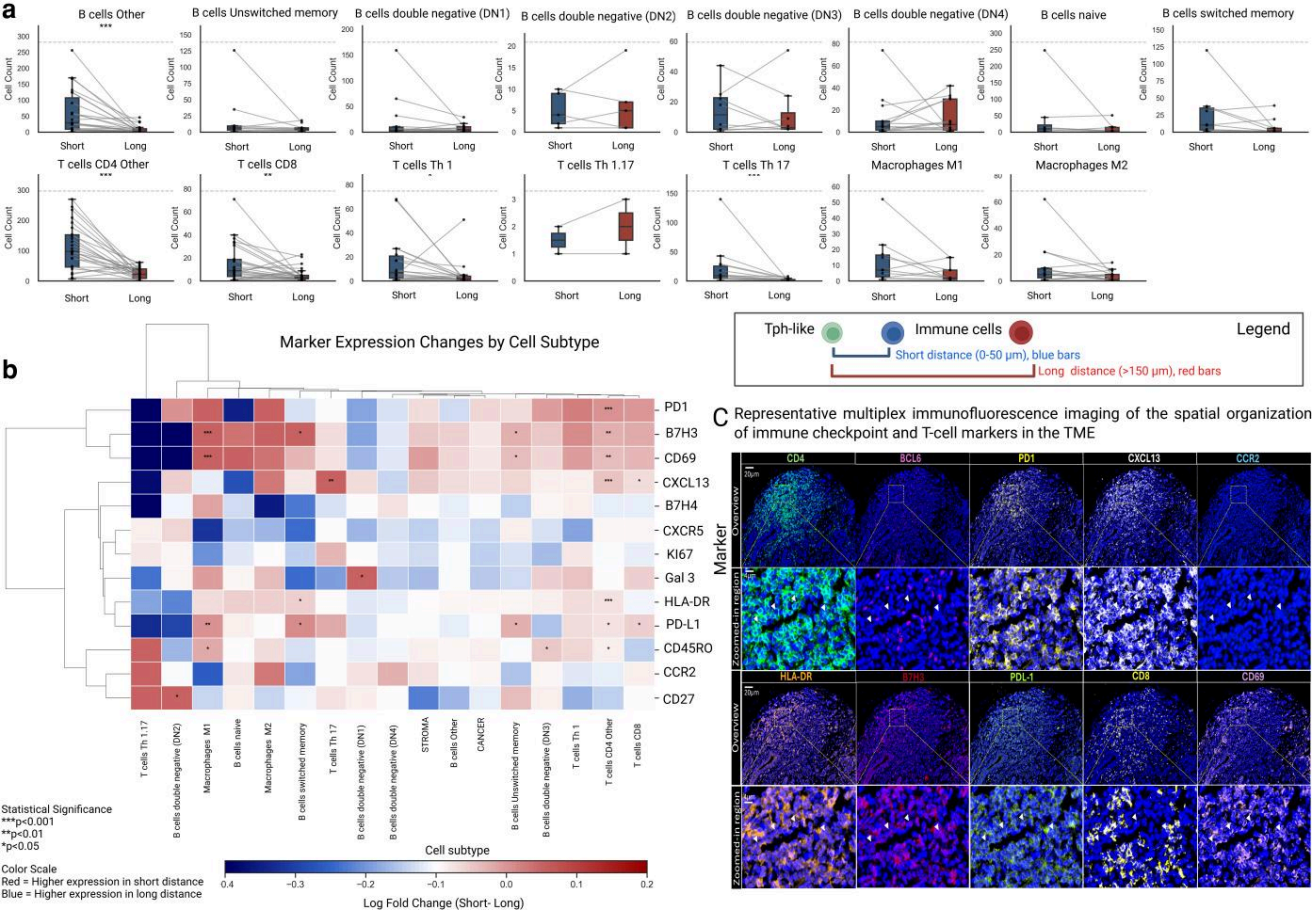
Spatial and phenotypic remodeling of immune cells across short- and long-interval lesions: modulation of marker expression in the vicinity of CCR2^−^ Tph-like cells. (a) Comparative spatial distribution of immune cell populations, showing box plots of cell counts within short (0–50 μm) and long (151–200 μm) intervals relative to Tph-like cells. Boxes represent the median and interquartile range, with whiskers extending to data points. (b) Heatmap visualization of spatially regulated marker expression, using CCR2^−^ Tph-like cells as the reference population. The color gradient represents log fold changes in marker expression between proximal (0–50 μm, red) and distal (151–200 μm, blue) regions. Statistical significance was determined using a one-sided Wilcoxon signed-rank test, comparing paired median marker expression values for each cell subtype per patient (* *P* < 0.05, ** *P* < 0.01, *** *P* < 0.001).(c) Multiplex immunofluorescence images illustrating the spatial organization of CCR2^−^ Tph-like cell niches within the tumor microenvironment in a breast cancer tissue biopsy. The top row highlights a CD4- and BCL6-low lymphoid aggregate exhibiting co-localized PD-1, CXCL13, and CCR2 expression, consistent with a CCR2^−^ Tph-like–enriched zone. The bottom row shows additional markers (HLA-DR, B7-H3, PD-L1, CD8, and CD69), revealing the presence of activated antigen-presenting cells and cytotoxic T cells distributed within and surrounding these aggregates, thereby validating the patterns observed in the heatmap analyses. Created in BioRender. Labrie, M. (2026) https://BioRender.com/eey2gn3.

Given that CCR2^−^ Tph-like cells exhibit a low CCR2 expression (Supplementary Figure S2), which suggests a tissue-resident profile, these results suggest a possible role of CCR2^−^ Tph-like cells in the recruitment of immune cells within the TME. Having a potential role in the recruitment of immune cells as a result of the expression of the chemoattractant CXCL13 (Fig. 2c), we would expect CCR2^−^ Tph-like cells to be in close proximity to immune cell populations, namely B cells and other TLS immune constituents. While TMAs enable high-throughput, efficient analysis across large numbers of samples, their limited sampling area poses a major limitation for reliable detection and characterization of TLS. As a result, subtle or spatially restricted TLS may be underrepresented or missed entirely. Given this, our primary objective was to leverage spatial single-cell analysis to characterize the cellular neighborhoods within each TMA core, specifically focusing on immune cell aggregation and the formation of immune niches surrounding CCR2^−^ Tph-like cells, rather than to comprehensively detect or quantify TLS presence. We utilized a Uniform Manifold Approximation and Projection (UMAP) plot to visualize the various immune cell populations in samples positive for Tph, Tfh, or CCR2^−^ Tph-like cells and found that while a few immune cells were included in the Tfh-positive samples, a very similar pattern of immune cell populations was observed between Tph and CCR2^−^ Tph-like (Fig. 3a). This could be explained by the fact that most CCR2^−^ Tph-like positive tissues are also positive for Tph cells. Interestingly, by coloring the cells based on their proximity to the nearest Tph, Tfh, or CCR2^−^ Tph-like cell, we observed that immune cells are markedly closer to CCR2^−^ Tph-like cells compared to Tph or Tfh cells (Fig. 3a). Specifically, the number of immune cells in close proximity to CCR2^−^ Tph-like cells is significantly higher within 100 μm radius around CCR2^−^ Tph-like cells compared to Tph and Tfh cells (Fig. 3c). Having identified the presence of immune clustering around CCR2^−^ Tph-like cells (Fig. 3a–c), we proceeded to characterize the specific immune populations and their marker profiles in the immediate surroundings of CCR2^−^ Tph-like cells. We compared these cells to more distal immune cells to identify immune subsets that preferentially locate near CCR2^−^ Tph-like cells and to assess potential differences in activation states, functional phenotypes, and immune interactions within the structured immune network. We conducted spatial analyses comparing immune populations at proximal (0–50 µm) and distal (151–200 µm) zones relative to CCR2^−^ Tph-like cells (Fig. 4a). These analyses revealed distinct organizational patterns, particularly within immune-enriched TMEs. Specifically, paired comparative analyses demonstrated significant spatial compartmentalization, with the proximal zones exhibiting significantly higher densities of multiple immune cell populations, including B cells, CD4^+^ T cells, CD8^+^ T cells, and Th1/Th17 subsets (Fig. 4a). This gradient-like distribution suggests a coordinated mechanism of immune cell positioning in which CCR2^−^ Tph-like cells reside at the core of immune aggregation sites, accompanied by additional immune subsets that likely participate in the same organized network.

Furthermore, single-cell spatial analysis confirmed a nonrandom immune architecture (Fig. 3a–c), demonstrating the preferential accumulation of key effector populations, within the immediate vicinity of CCR2^−^ Tph-like cells. These findings support the idea that CCR2 downregulation and the dense immune clustering around these cells may be mutually reinforcing processes reflecting their specialized role within inflamed TME regions.

### Immune populations within CCR2^−^ Tph-like cells’ immediate vicinity have a distinct immune signature

CCR2^−^ Tph-like cells appear to be associated with immune cell recruitment within the TME, but the molecular mechanisms governing their potential interactions with surrounding immune populations are unknown. To explore potential differences in immune cell phenotypes based on spatial proximity to CCR2^−^ Tph-like cells and to identify which populations were selectively enriched within these immune niches, we conducted a hierarchical clustering analysis of differential marker expression between immune cell populations from CCR2^−^ Tph-like proximal (0–50 µm) and distal (151–200 µm) regions. We compared cell densities in the proximal and distal regions, restricting the analysis to samples with matching cell populations in both areas. This analysis identified three distinct molecular profiles (Fig. 4b and c). Compared to distal regions, B7-H3 and CD69 expression in the proximal regions was markedly reduced in B-cell double negative (DN2) populations (*P* < 0.001) but significantly elevated in M1 macrophages (*P* < 0.001), CD8^+^ T cells (*P* < 0.01), and unswitched memory B cells (*P* < 0.05), indicating localized immune activation. Additionally, B7-H4 was significantly downregulated in DN2 B cells, suggesting a distinct regulatory state within this subset. PD-L1 was upregulated in M1 macrophages and CD4^+^ T cells (*P* < 0.05), while CD45RO and human leukocyte antigen—DR isotype (HLA-DR) were highly expressed in CD4^+^ T cells (*P* < 0.001), indicative of an antigen-experienced phenotype. Notably, HLA-DR and CD45RO expression were also increased in specific B-cell subtypes (*P* < 0.05), suggesting potential activation. Although PD-L1 expression was elevated in cancer cells, this increase did not reach statistical significance, implying a possible role in immune evasion. CXCL13 and PD-1 were strongly enriched in CD4^+^ T cells (*P* < 0.001) from the proximal region compared to distal, particularly within CXCL13-producing subsets, underscoring their involvement in immune recruitment and spatial immune organization. Furthermore, CCR2 and CXCR5 showed low expression in CD4^+^ T cells, CD8^+^ T cells, and B cells, suggesting a reduced migratory capacity and tissue retention in these populations. In the proximal compared to distal regions, Galectin-3 (Gal-3) was significantly upregulated in M2 macrophages (*P* < 0.05), separating them from M1 macrophages, which exhibited elevated PD-L1 expression. This distinction highlights functionally distinct macrophage subsets, potentially reflecting pro-inflammatory (M1) versus immunosuppressive (M2) roles. Although not statistically significant, those macrophages also exhibited lower levels of HLA-DR, suggesting differences in antigen presentation compared to T cells. Taken together, these findings suggest a spatially structured immune landscape, where specific immune populations are preferentially retained in the immune cluster near CCR2^−^ Tph-like cells potentially reflecting their involvement in shared immune networks. While further investigation is needed to confirm the precise role of CCR2^−^ Tph-like cells, these results provide insights into immune organization within the TME and highlight potential targets for immunotherapeutic strategies.

## Discussion

The main objective of this study was to assess the presence of Tph cells across cancer types and determine their spatial distribution and interaction with other immune cells within the TME. Using Cyc-IF we identified several CXCL13-positive CD4 T-cell populations, including Tph and Tfh cells. These cells can attract CXCR5+ cells, promoting the immune organization of the TME. Surprisingly, Cyc-IF analysis also led to identifying of a CXCL13+ CCR2^−^ cells that strongly correlated with tumor immune infiltration, consistent with an immunotherapy-responsive TME profile. The principal distinction observed between Tph and Tph-like cells in our study was the absence of CCR2 expression in the Tph-like subset. However, the lack of CCR2 alone is not sufficient to define CCR2^−^ Tph-like cells as a distinct lineage. Rather, this phenotypic variation may reflect differences in activation state, tissue adaptation, or microenvironmental cues within the broader Tph continuum. Further experimental investigation would be necessary to determine whether CCR2^−^ Tph-like cells represent a stable lineage or a transient differentiation state. CCR2 is a chemokine receptor associated with migrating immune cells to inflammatory sites, in response to its various ligands (CCL2, CCL7, CCL8, CCL13, CCL16, and PSMP) [31]. The absence of CCR2 in T cells might reduce their responsiveness to pro-chemoattractant molecules, which could favor their local persistence within the TME [32]. As Tph cells need CCR2 expression for peripheral circulation and homing to inflamed tissues, CCR2^−^ Tph-like cells may represent a subset of locally adapted, long-lived Tph cells contributing to sustaining immune responses within tumors. In other words, we propose these Tph-like cells to be functionally active resident Tph cells, derived from circulating Tph cells that have infiltrated and colonized the tumors.

CCR2^−^ Tph-like cells emerged as the predominant CXCL13+ CD4 T cell population in our cancer patient cohort, representing not only the most frequently observed phenotype but also demonstrating significantly higher density values compared to classic Tfh and Tph cell populations. The decreased CCR2 expression in CCR2^−^ Tph-like cells suggests that, once in the tissue, Tph-like cells lose their egress capacity and organize the formation of local ectopic germinal center analogous to the role of Tfh in lymph nodes. Indeed, while CCR2 downregulation typically impacts cellular migration to inflammatory sites [33], these CCR2^−^ Tph-like cells maintain their ability to produce CXCL13 (Supplementary Figure S2c and d), thereby preserving their capacity to orchestrate the local immune responses by influencing CXCR5+ cells. They principally act through B-cell recruitment and activation. The dominant prevalence of functional CCR2^−^ Tph-like cells, in TME, suggests these cells may play a previously unrecognized role in coordinating antitumor immunity. This finding underscores the need for functional investigations, including chemotaxis assays to assess directed migration toward specific chemokines, co-culture experiments to study cell–cell interactions and functional crosstalk, and perturbation studies involving gene knockdown or blockade of signaling pathways. Such approaches will help clarify the mechanistic roles of Tph-like cells in tumor immunity and inform the rational development of therapeutic strategies targeting tumor-associated immune responses. Indeed, Tfh and Tph cells play crucial roles in immune organization, particularly through their involvement in TLS formation and maturation in non-lymphoid tissues during chronic inflammation. While Tph cells secrete CXCL13 chemokine to attract B cells and promote TLS formation, Tfh cells can still support B-cell activation within the TLS [14]. This mechanism has been well documented in autoimmune diseases and is increasingly recognized in cancer, where TLS presence within the TME is linked to improved prognoses and enhanced immunotherapy responses [5]. Emerging evidence suggests that Tph cells may also play a critical role in TLS formation within tumors, contributing to local immune activation and antitumor immunity [15]. Nonetheless, due to the use of a tissue microarray, the limited sample size did not allow quantification of TLs. However, the expression of CXCL13 contributes to immune cell aggregation and the formation of ectopic lymphoid structures within inflamed tissues, reinforcing the organization of local immune responses [34]. Our analysis revealed a high concentration of B cells, CD4+ T cells, and CD8+ T cells within the CCR2^−^ Tph-like positive immune niches, suggesting a dynamic immune microenvironment for Tph. These lymphoid aggregates might represent early stages of tertiary TLS development [35]. A future study on whole tissue slides would be necessary to allow detection and quantification of TLS in samples infiltrated by CCR2^−^ Tph-like cells and confirm their correlation with TLS.

The spatial architecture and cell phenotypic analysis of the TME revealed organized patterns of immune cell distribution and composition. Building upon the established TME classification system proposed by Alexander *et al*, we focused on the previously described ‘Immune enriched’ phenotype, which is associated with improved patient outcomes across multiple cancer types [22]. Using this favorable TME classification, our analysis demonstrated a predominant presence of CCR2^−^ Tph-like cells compared to conventional Tfh and Tph populations. The spatial analysis revealed that only CCR2^−^ Tph-like cells exhibit enriched immune cell clustering in their vicinity, while Tfh and Tph cells showed no distinct aggregation patterns in their proximity. In zones enriched in CCR2^−^ Tph-like cells, we observed significantly higher densities of multiple immune cell populations than in distal regions. The co-localization of Th1 and Th17 cells with Tph-like cells indicates an active and polarized type 1 and type 3 immune responses, possibly driven by the local secretion of TNF and IL-6, key cytokines produced by these Th subsets. Th1 and Th17 cells are known to promote pro-inflammatory and antitumor responses [36]. Moreover, pro-inflammatory cytokines like TNF and IL-6 indeed play a crucial role in supporting the long-term production of CXCL13 by memory CD4^+^ T cells [37, 38]. Additionally, CCR2^−^ Tph-like cells exhibit high PD-1 expression, a hallmark of T-cell memory and chronic activation. These findings also suggest that anti–PD-1 immunotherapies may exert direct effects on CCR2^−^ Tph-like cells. However, while studies in other contexts suggest that Tfh and Tph populations can respond to checkpoint inhibition and may contribute to antitumor immunity, further research is necessary to determine whether anti-PD-1 therapy directly impacts Tph-like cells and to clarify the extent of their involvement in clinical responses.

Using CCR2^−^ cells as a reference point, a specific immunological niche characterized by upregulation of immune checkpoint molecules B7H3 (CD276) and CD69 was identified. These markers were upregulated in M1 macrophages, unswitched memory B cells, and total CD4 T cells, suggesting local regulation of the immune response and activation of both the cellular and humoral response. For unswitched B cells and CD4 T cells, the upregulation of B7H3 and CD69 implies a state of immunological activation that can be both protective and potentially suppressive [39, 40]. B7H3 may indeed function as an immunomodulatory molecule that fine-tunes cellular responses, like the other members of the B7 family, such as B7-1 (CD80) and B7-2 (CD86) that are expressed by various tumors and favor immune evasion [41]. B7H3 is thus potentially preventing uncontrolled immune activation while nonetheless inadvertently supporting tumor immune evasion. The immunosuppressive role of B7H3 in tumor has been recognized and even led to the development of Enoblituzumab, an anti-B7H3, as putative immunotherapy for solid cancer [42]. The CD69 marker’s presence indicates active cellular engagement, suggesting these populations are primed for potential immune intervention [43]. Additionally, CD69 downregulates S1P1 (sphingosine-1-phosphate receptor 1), preventing lymphocyte egress from lymphoid organs into circulation, *de facto* trapping these ‘activated cells’ in the TME [44]. The simultaneous increase in both markers across these cell types suggests a highly active immune system, potentially driven by tumor-associated antigens (TAAs) or excess release of damage-associated molecular patterns (DAMP) as a result of local antitumor activity. TAAs, arising from genetic mutations, aberrant protein expression, or post-translational modifications, are recognized by the immune system and can lead to chronic T-cell activation [45]. Persistent exposure to TAAs within the TME may result in sustained immune cell retention and activation, as reflected by CD69 upregulation. This prolonged immune engagement could either enhance antitumor immunity by maintaining effector T cells within the TME or, alternatively, drive immune exhaustion, depending on the balance between co-stimulatory and inhibitory signals [46]. While PD-1 expression is often linked to T-cell exhaustion in chronic antigen exposure settings, emerging evidence indicates that PD-1^+^ T cells, particularly in peripheral tissues, can remain functionally active and continue to participate in immune responses, despite high PD-1 expression [47]. Understanding how CCR2^−^ Tph-like cells maintain their functional activity regardless of PD-1 upregulation could provide valuable insights into overcoming immune checkpoint resistance and optimizing cancer immunotherapy strategies.

Finally, our analysis revealed a spatially coordinated immune response in proximal regions, characterized by the synchronized expression of PD-L1, CD45RO, and HLA-DR across CD4^+^ T cells and M1 macrophages. This molecular profile suggests functional crosstalk between memory and effector CD4^+^ T cells and antigen-presenting macrophages near Tph-like cells, potentially shaping local immune dynamics. The co-expression of CD45RO on CD4^+^ T cells confirms a memory/effector phenotype, indicating that these antigen-experienced cells are primed for rapid immune responses while simultaneously being subjected to regulation through local PD-L1-mediated immune modulation to prevent excessive inflammation. Meanwhile, PD-L1 upregulation in M1 macrophages implies a role for innate immunity in modulating T-cell activation within the TME, possibly contributing to mechanisms of peripheral immune tolerance and damping the adaptive response in the cancer microenvironment. Additionally, the downregulation of chemokine receptors CCR2 and CXCR5 in CD4^+^ T cells and B cells indicates that, after homing to tissues, these cells lose their ability to egress in response to their respective ligands, CCL2 and CXCL13. Combined with the expression of CD69^+^ immune cells near CCR2^−^ Tph-like cells, the loss of CCR2 and CXCR5 may further enhance local immune cell interactions. The presence of CXCL13-producing PD-1^+^ CD4^+^ T cells in these regions highlights their role as potential pioneer cells for the adaptive immune response with the potential to orchestrate immune cell recruitment, spatial organization, and antitumor immune responses. This suggests that Tph-like cells may act as key architects of intratumoral immune niches, influencing both protective immunity and immune regulation in cancer.

## Conclusion

Our findings suggest that CCR2^−^ Tph-like cells represent a previously unrecognized tissue-resident CD4^+^ T-cell subset with a potential role in shaping the immune architecture of the TME. Their high CXCL13 secretion, PD-1 expression, and spatial organization within immune-enriched niches suggest they may be key orchestrators of intratumoral immune responses, particularly in TLS formation. Moreover, their low CCR2 expression, indicative of potential reduced migratory capacity, raises important questions regarding their long-term persistence within tumors and their contribution to immune modulation in cancer. Further research will be necessary to monitor the expansion of CCR2^−^ Tph-like cells during immunotherapy, particularly in relation to immune checkpoint blockade responses and resistance mechanisms. Additionally, understanding the antigenic triggers that drive CCR2^−^ Tph-like cell activation, especially considering their high PD-1 expression and tissue-resident phenotype, will be crucial for determining their functional significance in tumor immunity. Investigating these aspects will not only enhance our understanding of CCR2^−^ Tph-like cell biology but may also provide novel therapeutic avenues for modulating the immune landscape in cancer.

## Supplementary Material

kyag007_Supplementary_Data

## Data Availability

All data will be available upon request.

## Abbreviations

ASHLAR: Alignment by Simultaneous Harmonization of Layer/Adjacency Registration
B7-H3: B7 homolog 3 protein
B7-H4: B7 homolog 4 protein
Bcl6: B-cell lymphoma 6 protein
CCR2: C-C motif chemokine receptor 2
CD69: early activation marker
CD45RO: memory T cell marker
CD4^+^ T cells: helper T cells
CD8^+^ T cells: cytotoxic T cells
CTLA-4: cytotoxic T-lymphocyte-associated protein 4
CXCL13: C-X-C motif chemokine ligand 13
CXCR5: C-X-C motif chemokine receptor 5
Cyc-IF: cyclic immunofluorescence
D HLA-DR: human leukocyte antigen—DR isotype (antigen presentation)
N2: B cell double negative 2 subset
irAEs: immune-related adverse events
ICI: immune checkpoint inhibitor
IQR: interquartile range
M1 macrophages: pro-inflammatory macrophages
M2 macrophages: immunosuppressive macrophages
PD-1: programmed cell death protein 1
PD-L1: programmed death-ligand 1
S1P1: sphingosine-1-phosphate receptor 1
SLAMF5: signaling lymphocytic activation molecule family member 5
TAA: tumor-associated antigen
Tfh: T follicular helper cell
Th1: T helper 1 cell
Th17: T helper 17 cell
TIGIT: T-cell immunoreceptor with Ig and ITIM domains
TLS: tertiary lymphoid structure
TMA: tissue microarray
TME: tumor microenvironment
Tph: T peripheral helper cell
Tph-like: T peripheral helper-like cell
UMAP: Uniform Manifold Approximation and Projection
