# Supplementary material for: CCR2− T peripheral helper cells as potential coordinators of local immune architecture in human cancer
Source: Discov Immunol. 2026 Mar 23;5(1):kyag007. doi: 10.1093/discim/kyag007 (PMC13058833; doi:10.1093/discim/kyag007)
Supplement: kyag007_Supplementary_Data [file kyag007_supplementary_data.zip › Supplementary Table I.docx]

| **Position** | **No.** | **Age** | **Sex** | **Organ/Anatomic Site** | **Pathology diagnosis** | **TNM** | **Grade** | **Stage** |
| --- | --- | --- | --- | --- | --- | --- | --- | --- |
| A1 | 1 | 61 | M | Stomach | Adenocarcinoma | T2N0M0 | 2 | IB |
| A2 | 2 | 56 | M | Stomach | Adenocarcinoma | T2N0M0 | 2 | IB |
| A3 | 3 | 60 | M | Stomach | Adenocarcinoma | T2N0M0 | 2 | IB |
| A4 | 4 | 60 | M | Stomach | Adenocarcinoma | T2N0M0 | 2--3 | IB |
| A5 | 5 | 66 | F | Stomach | Adenocarcinoma | T3N0M0 | 2--3 | IIA |
| A6 | 6 | 67 | M | Stomach | Adenocarcinoma | T2N0M0 | 3 | IB |
| A7 | 7 | 85 | M | Stomach | Adenocarcinoma | T2N0M0 | 3 | IB |
| A8 | 8 | 40 | M | Stomach | Adenocarcinoma | T4N0M0 | 3 | IIB |
| A9 | 9 | 61 | M | Esophagus | Adenocarcinoma | T3N0M0 | 2 | IIB |
| A10 | 10 | 58 | F | Esophagus | Adenocarcinoma | T4N0M0 | 3 | IIIA |
| A11 | 11 | 69 | F | Esophagus | Adenocarcinoma | T3N1M0 | 3 | IIIA |
| A12 | 12 | 33 | F | Esophagus | Adenocarcinoma | T3N0M0 | 3 | IIB |
| A13 | 13 | 54 | F | Esophagus | Squamous cell carcinoma | T4aN2M0 | 1 | IIIC |
| A14 | 14 | 62 | M | Esophagus | Squamous cell carcinoma | T3N1M0 | 2 | IIIA |
| A15 | 15 | 56 | M | Esophagus | Squamous cell carcinoma | T3N0M0 | 2 | IIA |
| A16 | 16 | 68 | M | Esophagus | Squamous cell carcinoma | T3N0M0 | 2 | IIA |
| B1 | 17 | 66 | M | Colon | Adenocarcinoma | T4N0M0 | 1 | IIB |
| B2 | 18 | 59 | M | Colon | Adenocarcinoma | T4N0M0 | 1--2 | IIB |
| B3 | 19 | 53 | M | Colon | Adenocarcinoma | T4N0M0 | 2 | IIB |
| B4 | 20 | 79 | F | Colon | Adenocarcinoma | T3N0M0 | 2 | IIA |
| B5 | 21 | 54 | M | Colon | Adenocarcinoma | T3N0M0 | 2 | IIA |
| B6 | 22 | 64 | M | Colon | Adenocarcinoma | T3N1M0 | 1 | IIIB |
| B7 | 23 | 58 | F | Colon | Adenocarcinoma | T4N0M0 | 2 | IIB |
| B8 | 24 | 53 | M | Colon | Adenocarcinoma | T3N0M0 | 2 | IIA |
| B9 | 25 | 33 | M | Liver | Hepatocellular carcinoma | T3N0M0 | 1 | IIIA |
| B10 | 26 | 46 | M | Liver | Hepatocellular carcinoma | T2N0M0 | 1--2 | II |
| B11 | 27 | 67 | F | Liver | Hepatocellular carcinoma | T1N0M0 | 2 | I |
| B12 | 28 | 35 | F | Liver | Hepatocellular carcinoma | T3N0M0 | 2 | IIIA |
| B13 | 29 | 19 | M | Liver | Hepatocellular carcinoma | T2N0M0 | 2 | II |
| B14 | 30 | 47 | M | Liver | Hepatocellular carcinoma | T2N0M0 | 2 | II |
| B15 | 31 | 75 | M | Liver | Hepatocellular carcinoma | T2N0M0 | 2--3 | II |
| B16 | 32 | 37 | M | Liver | Hepatocellular carcinoma | T2N0M0 | 2--3 | II |
| C1 | 33 | 68 | F | Pancreas | Adenocarcinoma | T2N0M0 | 2 | IB |
| C2 | 34 | 45 | M | Pancreas | Duct adenocarcinoma | T2N0M0 | 2 | IB |
| C3 | 35 | 55 | M | Pancreas | Duct adenocarcinoma | T3N0M0 | 2 | IIA |
| C4 | 36 | 44 | M | Pancreas | Adenocarcinoma | T3N0M0 | 2 | IIA |
| C5 | 37 | 49 | M | Pancreas | Duct adenocarcinoma | T2N0M0 | 2 | IB |
| C6 | 38 | 54 | F | Pancreas | Duct adenocarcinoma | T2N0M0 | 2 | IB |
| C7 | 39 | 54 | M | Pancreas | Duct adenocarcinoma | T3N0M0 | 2 | IIA |
| C8 | 40 | 23 | F | Pancreas | Duct adenocarcinoma | T3N0M0 | * | IIA |
| C9 | 41 | 66 | M | Lung | Squamous cell carcinoma | T2N0M0 | 1 | IB |
| C10 | 42 | 56 | M | Lung | Squamous cell carcinoma | T3N0M0 | 2--3 | IIB |
| C11 | 43 | 42 | F | Lung | Papillary adenocarcinoma | T3N1M0 | 2--3 | IIIA |
| C13 | 45 | 47 | F | Lung | Large cell carcinoma | T2N2M0 | * | IIIA |
| C14 | 46 | 64 | M | Lung | Large cell carcinoma | T2N0M0 | - | IB |
| C15 | 47 | 73 | M | Lung | Small cell carcinoma | - | - | - |
| C16 | 48 | 50 | M | Lung | Small cell carcinoma | - | - | - |
| E1 | 65 | 36 | F | Thyroid gland | Papillary adenocarcinoma | T3N0M0 | - | I |
| E2 | 66 | 60 | F | Thyroid gland | Papillary adenocarcinoma | T2N0M0 | - | II |
| E3 | 67 | 65 | F | Thyroid gland | Papillary adenocarcinoma | T2N0M0 | - | II |
| E4 | 68 | 48 | F | Thyroid gland | Papillary adenocarcinoma | T3N0M0 | - | III |
| E5 | 69 | 63 | M | Thyroid gland | Follicular carcinoma | T3N0M0 | - | III |
| E6 | 70 | 36 | F | Thyroid gland | Follicular carcinoma | T3N0M0 | - | I |
| E7 | 71 | 43 | F | Thyroid gland | Follicular carcinoma | T2N0M0 | - | I |
| E8 | 72 | 29 | F | Thyroid gland | Follicular carcinoma | T1N0M0 | - | I |
| F1 | 81 | 49 | M | Cheek | Squamous cell carcinoma of left cheek | T1N0M0 | 2 | I |
| F2 | 82 | 49 | M | Gingiva | Squamous cell carcinoma | T4N0M0 | 3 | IV |
| F3 | 83 | 57 | F | Cheek | Squamous cell carcinoma of left cheek | T3N0M0 | 1 | III |
| F4 | 84 | 56 | F | Oral cavity | Squamous cell carcinoma of mouth floor | T2N0M0 | 3 | II |
| F5 | 85 | 63 | F | Soft palate | Squamous cell carcinoma | - | 2 | - |
| F6 | 86 | 63 | M | Nose | Squamous cell carcinoma of right nasal cavity | T1N0M0 | 1 | I |
| F7 | 87 | 43 | M | Nose | Squamous cell carcinoma of left nasal cavity | T3N0M0 | 3 | III |
| F8 | 88 | 47 | F | Maxillary sinus | Squamous cell carcinoma | T1N0M0 | 2--3 | I |
| F9 | 89 | 53 | M | Lip | Squamous cell carcinoma of upper lip | T1N0M0 | 1 | I |
| F10 | 90 | 63 | M | Skin | Squamous cell carcinoma of crissum | T3N0M0 | 1 | III |
| F11 | 91 | 80 | F | Skin | Squamous cell carcinoma of right face | T2N0M0 | 1 | II |
| F12 | 92 | 72 | M | Skin | Squamous cell carcinoma of buttock | T2N0M0 | 1 | II |
| F13 | 93 | 60 | M | Skin | Squamous cell carcinoma of buttock | T3N0M0 | 1 | III |
| F14 | 94 | 68 | M | Skin | Squamous cell carcinoma of left temporal part | T2N0M0 | 1 | II |
| F15 | 95 | 62 | M | Skin | Squamous cell carcinoma of occipitalia part | T2N0M0 | 1 | I |
| F16 | 96 | 82 | F | Skin | Squamous cell carcinoma of right thumb | T1N0M0 | 2--3 | I |
| G1 | 97 | 32 | M | Skin | Dermatofibrosarcoma protuberans of left chest wall | T2aN0M0 | - | IB |
| G2 | 98 | 15 | M | Skin | Dermatofibrosarcoma protuberans of right waist part | T2aN0M0 | - | IB |
| G3 | 99 | 58 | M | Skin | Dermatofibrosarcoma of left dorsum of foot | T2aN0M0 | - | IB |
| G4 | 100 | 58 | F | Skin | Dermatofibrosarcoma protuberans of chest wall | T1aN0M0 | - | IA |
| G5 | 101 | 72 | M | Skin | Atypical liposarcoma of right thigh | T1N0M0 | - | IA |
| G6 | 102 | 68 | F | Skin | Myxoid liposarcoma of waist and back | T1aN0M0 | - | IIA |
| G8 | 104 | 40 | F | Skin | Myxoid liposarcoma of left thigh | T2aN0M0 | - | IIB |
| G9 | 105 | 53 | M | Skin | Malignant melanoma of sole of foot | T4N0M0 | - | IIB |
| G10 | 106 | 42 | M | Skin | Malignant melanoma of left heel of foot | T3N0M0 | - | IIA |
| G11 | 107 | 45 | F | Skin | Malignant melanoma of left chest wall | T4N0M0 | - | IIB |
| G12 | 108 | 77 | F | Skin | Malignant melanoma of sole of foot | T4N0M0 | - | IIB |
| G13 | 109 | 52 | M | Skin | Malignant melanoma of crissum | T3N0M0 | - | IIA |
| G14 | 110 | 74 | F | Skin | Malignant melanoma of back | T4aN0M0 | - | IIB |
| G15 | 111 | 66 | M | Rectum | Malignant melanoma | - | - | - |
| G16 | 112 | 67 | F | Skin | Malignant melanoma of crissum | T4N0M0 | - | IIB |
| H1 | 113 | 65 | F | Breast | Invasive carcinoma of no special type | T2N0M0 | 1--2 | IIA |
| H2 | 114 | 37 | - | Breast | Invasive carcinoma of no special type | T2N0M0 | 1--2 | IIA |
| H3 | 115 | 71 | F | Breast | Invasive carcinoma of no special type | T2M2N0 | 2 | IIIA |
| H4 | 116 | 38 | F | Breast | Invasive carcinoma of no special type | T1N1M0 | 2 | IIA |
| H5 | 117 | 46 | F | Breast | Invasive carcinoma of no special type | T2N0M0 | 2 | IIA |
| H6 | 118 | 63 | F | Breast | Invasive carcinoma of no special type | T2N0M0 | 2 | IIA |
| H7 | 119 | 30 | F | Breast | Invasive carcinoma of no special type | T1N0M0 | 2 | IA |
| H8 | 120 | 65 | F | Breast | Invasive carcinoma of no special type | T1N2M0 | 3 | IIIA |
| H9 | 121 | 35 | F | Breast | Invasive lobular carcinoma | T3N0M0 | - | IIB |
| H10 | 122 | 37 | F | Breast | Invasive lobular carcinoma | T4N1M0 | - | IIIB |
| H11 | 123 | 59 | F | Breast | Invasive lobular carcinoma | T4N0M0 | - | IIIB |
| H12 | 124 | 49 | F | Breast | Invasive lobular carcinoma | T2N0M0 | - | IIA |
| H13 | 125 | 51 | F | Breast | Invasive lobular carcinoma | T2N1M0 | - | IIB |
| H14 | 126 | 45 | F | Breast | Invasive lobular carcinoma | T2N0M0 | - | IIA |
| H15 | 127 | 70 | F | Breast | Invasive lobular carcinoma | T4N0M0 | - | IIIB |
| H16 | 128 | 70 | F | Breast | Invasive lobular carcinoma | T2N2M0 | - | IIIA |
| I2 | 130 | 41 | F | Ovary | Low grade serous papillary carcinoma | T1aN0M0 | - | IA |
| I3 | 131 | 73 | F | Ovary | High grade serous carcinoma | T1bN0M0 | 3 | IB |
| I4 | 132 | 57 | F | Ovary | High grade serous carcinoma | T2bN0M0 | 2--3 | IIB |
| I5 | 133 | 66 | F | Ovary | High grade serous carcinoma | T3aN0M0 | 2 | IIIA |
| I6 | 134 | 57 | F | Ovary | High grade serous carcinoma | T1cN0M0 | 3 | IC |
| I7 | 135 | 55 | F | Ovary | High grade serous carcinoma | T1aN0M0 | 3 | IA |
| I8 | 136 | 56 | F | Ovary | High grade serous carcinoma | T2N0M0 | 3 | II |
| I13 | 141 | 46 | F | Ovary | Sertoli-Leydig cell tumor | - | - | - |
| I14 | 142 | 40 | F | Ovary | Moderately differentiated sertoli-leydig cell tumor | - | - | - |
| I15 | 143 | 59 | F | Ovary | Moderately differentiated sertoli cell tumor | - | - | - |
| I16 | 144 | 26 | F | Ovary | Poorly differentiated sertoli-leydig cell tumor | - | - | - |
| J1 | 145 | 40 | F | Uterus | Endometrioid adenocarcinoma | T2N0M0 | 1 | II |
| J2 | 146 | 65 | F | Uterus | Endometrioid adenocarcinoma | T1aN0M0 | 1 | I |
| J3 | 147 | 53 | F | Uterus | Endometrioid adenocarcinoma | T1bN0M0 | 1 | IB |
| J4 | 148 | 48 | F | Uterus | Endometrioid adenocarcinoma | T2N0M0 | 1--2 | II |
| J5 | 149 | 50 | F | Uterus | Endometrioid adenocarcinoma | T2N0M0 | 2 | II |
| J6 | 150 | 57 | F | Uterus | Endometrioid adenocarcinoma | T1bN0M0 | 2 | IB |
| J7 | 151 | 64 | F | Uterus | Endometrioid adenocarcinoma | T1bN0M0 | 3 | IB |
| J8 | 152 | 65 | F | Uterus | Endometrioid adenocarcinoma | T1bN0M0 | 1 | IB |
| J9 | 153 | 62 | F | Cervix | Squamous cell carcinoma | T1N0M0 | 1 | I |
| J10 | 154 | 49 | F | Cervix | Squamous cell carcinoma | T1N0M0 | 1 | I |
| J11 | 155 | 38 | F | Cervix | Squamous cell carcinoma | T1bN0M0 | 2 | IB |
| J12 | 156 | 41 | F | Cervix | Squamous cell carcinoma | T1N0M0 | 2 | I |
| J13 | 157 | 37 | F | Cervix | Squamous cell carcinoma | T1N0M0 | 2--3 | I |
| J14 | 158 | 49 | F | Cervix | Squamous cell carcinoma | T1N0M0 | 2--3 | I |
| J15 | 159 | 55 | F | Cervix | Squamous cell carcinoma | T2aN0M0 | 3 | IIA |
| J16 | 160 | 29 | F | Cervix | Squamous cell carcinoma | T1N0M0 | 3 | I |
| K1 | 161 | 66 | M | Prostate | Adenocarcinoma (Gleason grade: 3, Gleason score: 3+3) | T2bN0M0 | 1 | IIA |
| K2 | 162 | 73 | M | Prostate | Adenocarcinoma (Gleason grade: 4, Gleason score: 3+4) | T2aN0M0 | 2 | IIA |
| K3 | 163 | 73 | M | Prostate | Adenocarcinoma (Gleason grade: 4, Gleason score: 3+4) | T2aN0M0 | 1 | IIA |
| K4 | 164 | 64 | M | Prostate | Adenocarcinoma (Gleason grade: 4, Gleason score: 4+4) | T2aN0M0 | 2 | IIA |
| K5 | 165 | 68 | M | Prostate | Adenocarcinoma (Gleason grade: 4, Gleason score: 3+4) | T2bN0M0 | 2 | IIA |
| K6 | 166 | 57 | M | Prostate | Adenocarcinoma (Gleason grade: 4, Gleason score: 3+4) | T2bN0M0 | 2 | IIA |
| K7 | 167 | 70 | M | Prostate | Adenocarcinoma (Gleason grade: 4, Gleason score: 4+3) | T2bN0M0 | 2--3 | IIA |
| K8 | 168 | 82 | M | Prostate | Adenocarcinoma (Gleason grade: 5, Gleason score: 5+5) | T3N0M0 | 3 | III |
| L1 | 177 | 70 | M | Kidney | Clear cell carcinoma | T1N0M0 | 1 | I |
| L2 | 178 | 46 | M | Kidney | Clear cell carcinoma | T1aN0M0 | 1 | I |
| L3 | 179 | 54 | M | Kidney | Clear cell carcinoma | T1N0M0 | 1 | I |
| L4 | 180 | 50 | M | Kidney | Clear cell carcinoma | T1N0M0 | 1 | I |
| L5 | 181 | 54 | M | Kidney | Clear cell carcinoma | T2N0M0 | 1 | II |
| L6 | 182 | 55 | M | Kidney | Clear cell carcinoma | T3N0M0 | 2 | III |
| L7 | 183 | 52 | F | Kidney | Clear cell carcinoma | T1bN0M0 | 2--3 | I |
| L8 | 184 | 41 | M | Kidney | Clear cell carcinoma | T2N0M0 | 2--3 | II |
| L9 | 185 | 48 | M | Bladder | High grade urothelial carcinoma | T1N0M0 | - | I |
| L10 | 186 | 45 | M | Bladder | High grade urothelial carcinoma | T3bN0M0 | - | IIIB |
| L11 | 187 | 74 | M | Bladder | High grade urothelial carcinoma | T2N0M0 | - | II |
| L12 | 188 | 67 | M | Bladder | High grade urothelial carcinoma | T1N0M0 | - | I |
| L13 | 189 | 36 | F | Bladder | High grade urothelial carcinoma | T2N0M0 | - | II |
| L14 | 190 | 55 | M | Bladder | High grade urothelial carcinoma | T3bN0M0 | - | IIIB |
| L15 | 191 | 50 | M | Bladder | High grade urothelial carcinoma | T3bN0M0 | - | IIIB |
| L16 | 192 | 62 | M | Bladder | High grade urothelial carcinoma | T1N0M0 | - | I |
