# Supplementary material for: CCR2− T peripheral helper cells as potential coordinators of local immune architecture in human cancer
Source: Discov Immunol. 2026 Mar 23;5(1):kyag007. doi: 10.1093/discim/kyag007 (PMC13058833; doi:10.1093/discim/kyag007)
Supplement: kyag007_Supplementary_Data [file kyag007_supplementary_data.zip › Supplementary.docx]

**SUPPLEMENTARY MATERIAL**

**SUPLEMENTARY FIGURE 1.** Expression of CCR2 in epithelial cells in human tonsil tissue.
Representative multiplex CyC-IF staining of FFPE human tonsil sections showing expression of CCR2 (cyan), Cytokeratin (magenta), and E-cadherin (orange), with nuclei counterstained using DAPI (blue). The merged image demonstrates co-localization of CCR2 with epithelial markers, consistent with expected expression patterns

**Supplementary Table I.** Pan-Cancer Tissue Microarray (TMA) Composition and Patient Information.

**SUPPLEMENTARY FIGURE 2.** Enrichment of CCR2⁻ Tph‑like cells in immune‑enriched TMEs and relationship between CXCL13 and CCR2 expression. (A) Distribution of tumors according to dominant helper T‑cell phenotype (Tph, Tph‑like, Tfh, combinations, or none) within each TME class (IE, IE/F, F, D), shown as the percentage of patients per group. (B) Paired comparisons of helper T‑cell phenotypes within tumors using a dominance index $\boldsymbol{\Delta}=(\boldsymbol{b}-\boldsymbol{c})/(\boldsymbol{b}+\boldsymbol{c})$, where $\boldsymbol{\Delta}>\mathbf{0}$ indicates dominance of the first‑listed phenotype; points indicate observed $\boldsymbol{\Delta}$, horizontal lines represent 95% Clopper–Pearson confidence intervals, and significance is assessed by two‑sided exact McNemar tests with Holm correction (ns, not significant; *, P < 0.05; **, P < 0.01; ***, P < 0.001; ****, P < 0.0001). (C,D) Violin/box plots of per‑cell CXCL13 (C) and CCR2 (D) intensity (log10[signal + 1]) across Tph, CCR2⁻ Tph‑like, Tfh and Treg subsets. CXCL13 expression is high in Tph, CCR2⁻ Tph‑like and Tfh cells but low in Treg cells, whereas CCR2 expression is selectively enriched in Tph cells and reduced in CCR2⁻ Tph‑like, Tfh and Treg cells; groups sharing the same letter are not significantly different by Kruskal–Wallis test with Dunn’s post‑hoc multiple‑comparison adjustment. Together, these analyses indicate that CXCL13 expression is broadly shared among helper T‑cell phenotypes and is largely independent of CCR2 expression, which specifically marks CCR2⁻ Tph-like cells.

**Supplementary Table II. Panel of Antibodies Utilized in the Cyc-IF Assay**
