# Supplementary material for: CCR2− T peripheral helper cells as potential coordinators of local immune architecture in human cancer
Source: Discov Immunol. 2026 Mar 23;5(1):kyag007. doi: 10.1093/discim/kyag007 (PMC13058833; doi:10.1093/discim/kyag007)
Supplement: kyag007_Supplementary_Data [file kyag007_supplementary_data.zip › Supplementry Table II.docx]

| **Marker** | **Description** | **Vendor** | **Catalog #** | **Dye** | **Clone** |
| --- | --- | --- | --- | --- | --- |
| CD4 | Recombinant Alexa Fluor® 647 Anti-CD4 antibody [EPR6855] (ab196147) | Abcam | ab196147 | AF647 | EPR6855 |
| CD44 | Anti-CD44 antibody | Abcam | ab157107 | AF750 | poly |
| CD45 | Alexa Fluor® 647 Anti-CD45 antibody [EP322Y] | Abcam | ab200317 | AF647 | EP322Y |
| CD68 | Purified anti-CD68 Antibody (KP1) (unconjugated) | Biolegend | 916104 | AF555 | KP1 |
| FOXP3 | Recombinant Alexa Fluor® 555 Anti-FOXP3 antibody [SP97] | Abcam | ab275120 | AF555 | SP97 |
| CK8 | Purified anti-Cytokeratin 8 | Biolegend | 904804 | AF750 | 1E8 |
| CK19 | Purified anti-Cytokeratin 19 | Biolegend | 628502 | AF750 | A53-B/A2 |
| Ecad | E-Cadherin Antibody, Rabbit Polyclonal | Sino biological | 50671-RP01 | AF750 | poly |
| aSMA | Recombinant Alexa Fluor® 488 Anti-alpha smooth muscle Actin antibody [EPR5368] | Abcam | ab202295 | AF488 | EPR5368 |
| CD31 | Anti-PECAM1 | St Johns Laboratory | STJ92117-100UL | AF750 | poly |
| Vimentin | Vimentin (D21H3) XP® Rabbit mAb (Alexa Fluor® 488 Conjugate) #9854 | Cell Signaling | 9854S | AF488 | D21H3 |
| ColVI | Recombinant Alexa Fluor® 488 Anti-Collagen VI antibody [EPR17072] (ab200429) | Abcam | ab200429 | AF488 | EPR17072 |
| Ki67 | Ki67/MKI67 Antibody [Alexa Fluor® 750] | Novus | NB110-90592 | AF750 | poly |
| CD163 | Alexa Fluor® 555 Anti-CD163 antibody [EPR19518] | Abcam | ab281746 | AF555 | EPR19518 |
| CD8 | CD8a Monoclonal Antibody (AMC908), eFluor™ 660, eBioscience™ | Life Tech | 50-0008-82 | AF647 | AMC908 |
| CD3 | CD3ε (D7A6E™) XP® Rabbit mAb (Alexa Fluor® 555 Conjugate) #57869 | CST | 57869S | AF555 | D7A6E |
| CD19 | CD19 Monoclonal Antibody (6OMP31), Alexa Fluor™ 488 | Invitrogen | 53-0194-82 | AF555 | 60MP31 |
| IgD | Goat anti-human IgD:FITC | BioRad | STAR143F | FITC | poly |
| Tbet | Recombinant AlexaFluor 647 Anti-T-bet / Tbx21 antibody [EPR9302] (ab225206) | Abcam | ab225206 | AF647 | EPR9302 |
| RORC | ROR gamma/RORC/NR1F3 Antibody (RORC/8017R) [DyLight 550] | Novus | NBP3-20738R | DyLight 550 | RORC/8017R |
| CXCL13 | CXCL13 Polyclonal Antibody | Invitrogen | PA5-28827 | AF647 | poly |
| CXCR5 | PE/Cyanine7 anti-human CD185 (CXCR5) Antibody | Biolegend | 356924 | PECy7 | J252D4 |
| CCR2 | CCR2 Recombinant Rabbit Monoclonal Antibody (SN707)(unconjugated) | ThermoFisher | MA5-41175 | AF647 | SN707 |
| BCL6 | Anti-Bcl6 antibody [BCL6/1527] | Abcam | ab218509 | AF647 | BCL6/1527 |
| CD11c | CD11c (D3V1E) XP® Rabbit mAb (Alexa Fluor® 647 Conjugate) #42756 | CST | 42756 | AF647 | D3V1E |
| PDL1 | Recombinant Alexa Fluor® 647 Anti-PD-L1 antibody [28-8] - Extracellular domain | Abcam | ab209960 | AF647 | 45166 |
| PD1 | Recombinant Alexa Fluor® 555 Anti-PD1 antibody [EPR4877(2)] (ab275126) | Abcam | ab275126 | AF555 | EPR4877(2) |
| GAL9 | Anti-galectin 9/Gal-9 antibody (ab69630) | Abcam | ab69630 | AF750 | poly |
| GAL3 | AlexaFluor 647 Anti-mouse/humain Mac-2 (galectin-3) | Biolegend | 125408 | AF647 | M3/38 |
| B7H3 | B7-H3/CD276 Antibody | Novus | NBP2-32251 | AF488 | poly |
| B7H4 | Anti-B7H4 antibody [EPR23665-20] (ab252438) | Abcam | ab252438 | AF555 | EPR23665-20 |
| CD27 | Anti-CD27 antibody (ab175403) | Abcam | ab175403 | AF488 | poly |
| CD45RO | Alexa Fluor® 488 anti-human CD45RO Antibody | Biolegend | 304212 | AF488 | UCHL1 |
| CD45RA | CD45RA antibody \| F8-11-13 | BioRad | MCA88A488 | AF488 | F8-11-13 |
| HLA-DR | Anti-HLA-DR antibody [HLA-Pan/2967R] | Abcam | ab257320 | AF488 | HLA-Pan/2967R |
| MHC class 1 | Anti-HLA Class 1 ABC antibody [EMR8-5] | Abcam | ab70328 | AF750 | EMR8-5 |
| TIM-3 | Purified anti-human CD366 (Tim-3) Antibody | BioLegend | 345002 | AF750 | F38-2E2 |
| Fibronectin | Anti-Fibronectin antibody [F14] (ab45688) monoclonal | Abcam | ab45688 | AF555 | F14 |
| Desmin | Recombinant Alexa Fluor® 488 Anti-Desmin antibody [Y66] - Cytoskeleton Marker | Abcam | ab185033 | AF488 | Y66 |
