# Supplementary figures and images for: CCR2− T peripheral helper cells as potential coordinators of local immune architecture in human cancer
Source: Discov Immunol. 2026 Mar 23;5(1):kyag007. doi: 10.1093/discim/kyag007 (PMC13058833; doi:10.1093/discim/kyag007)

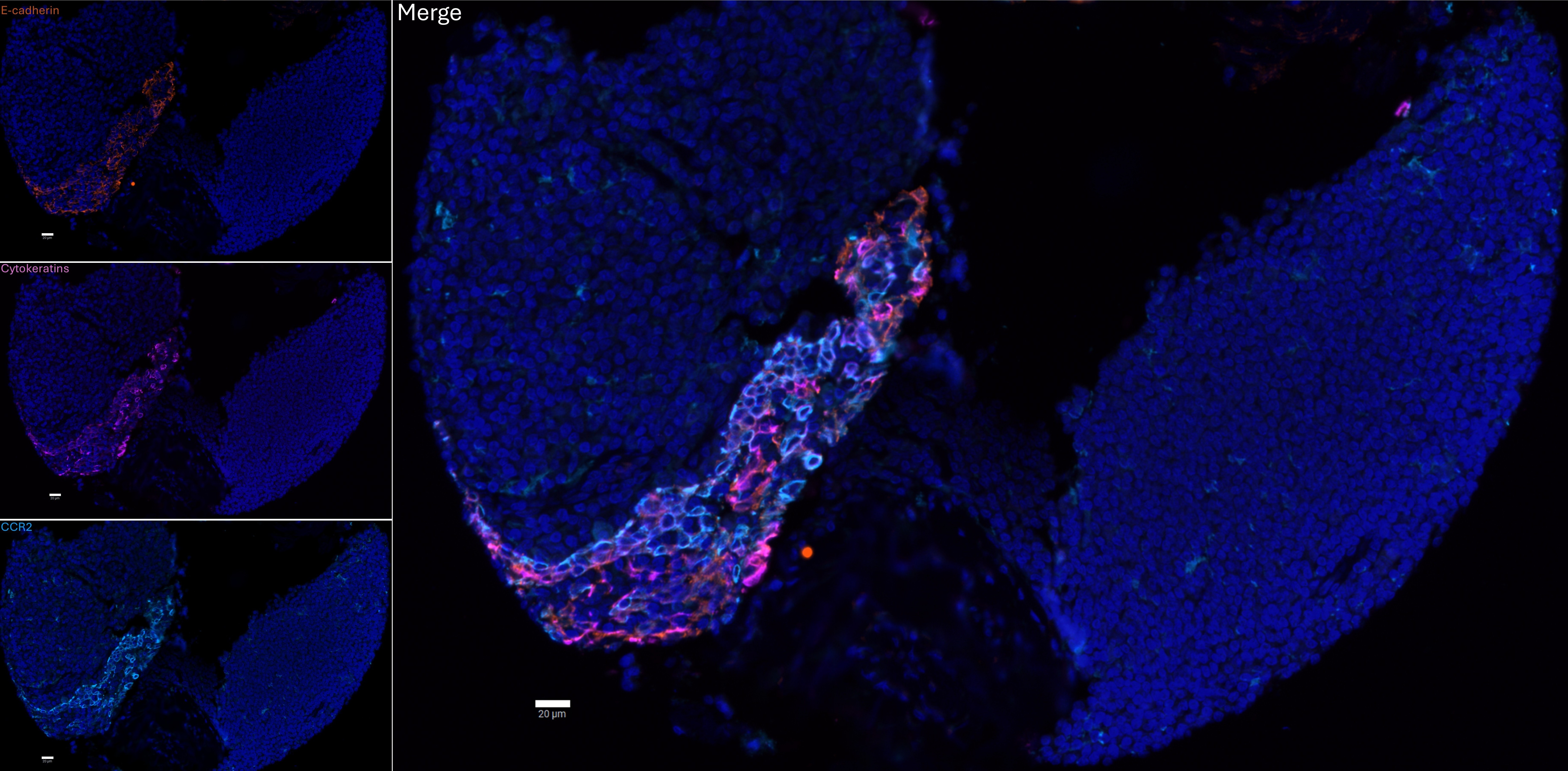

Supplement: kyag007_Supplementary_Data [file kyag007_supplementary_data.zip › Figure 1S.jpg]

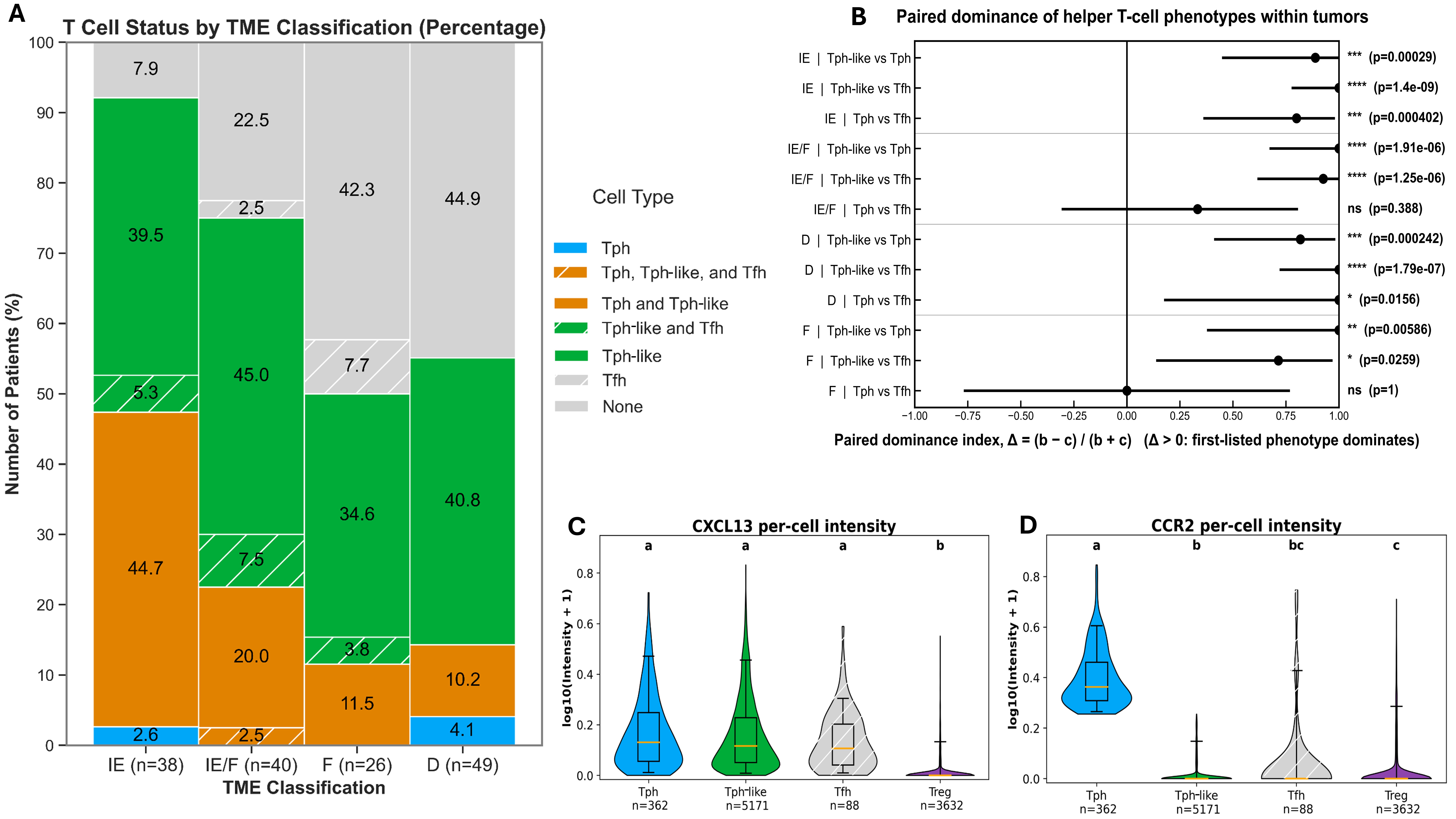

Supplement: kyag007_Supplementary_Data [file kyag007_supplementary_data.zip › Figure S2.jpg]
